# A deeper look into natural sciences with physics-based and data-driven measures

**DOI:** 10.1016/j.isci.2021.102171

**Published:** 2021-02-09

**Authors:** Davi Röhe Rodrigues, Karin Everschor-Sitte, Susanne Gerber, Illia Horenko

**Affiliations:** 1 Institute of Physics, Johannes Gutenberg University of Mainz, 55128 Mainz, Germany; 2 Institute of Human Genetics, University Medical Center of the Johannes Gutenberg University Mainz, 55131 Mainz, Germany; 3 Università della Svizzera Italiana, Faculty of Informatics, Via G. Buffi 13, 6900 Lugano, Switzerland

**Keywords:** Physics, Magnetism, Applied Physics, Computer Science, Artificial Intelligence

## Abstract

With the development of machine learning in recent years, it is possible to glean much more information from an experimental data set to study matter. In this perspective, we discuss some state-of-the-art data-driven tools to analyze latent effects in data and explain their applicability in natural science, focusing on two recently introduced, physics-motivated computationally cheap tools—latent entropy and latent dimension. We exemplify their capabilities by applying them on several examples in the natural sciences and show that they reveal so far unobserved features such as, for example, a gradient in a magnetic measurement and a latent network of glymphatic channels from the mouse brain microscopy data. What sets these techniques apart is the relaxation of restrictive assumptions typical of many machine learning models and instead incorporating aspects that best fit the dynamical systems at hand.

## Graphical abstract

**Figure:**
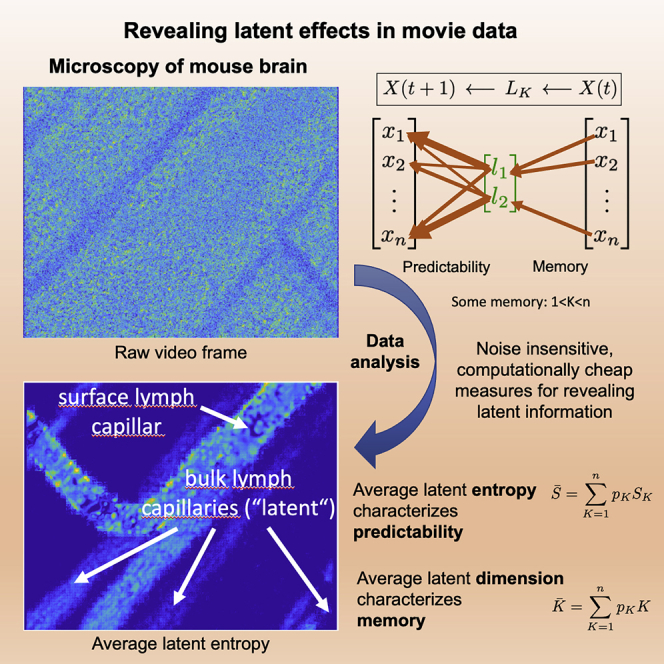

## Introduction

Being able to explore further length scales than those accessible for the human eye (limited to the range of millimeters to kilometers) allowed for a better understanding of natural sciences. Experimental techniques have evolved significantly to explore the different space scales, from new generation electron microscopes to deep-field telescopes. However, a great challenge imposed by increasing experimental instruments' sensitivity is the excess of information that must be filtered to study the desired behavior. For this reason, there is great demand for a simultaneous development of computational machine learning (ML) techniques to analyze data. The understanding of patterns hidden in the noise has been awarded at least two Physics Nobel Prizes—the discovery of cosmic microwave background (Penzias and Wilson, 1965) in 1978 and gravitational waves (Abbott et al., 2017) in 2017—and lead, for example, to the first image of a black hole (The Event Horizon Telescope Collaboration, 2019a) in 2019. These recent successes rely enormously on the progress achieved in data inference and ML techniques.

While ML has progressed substantially within the last decades, being used across all fields of natural sciences and becoming part of our daily life, the field still faces several challenges. We emphasize two challenges: (i) the computational costs, which still go hand in hand with the ever-increasing size of the data sets, and (ii) often assumed properties restricting the data analysis, such as independency and identically distribution of the data (Blum and Langley,1997; Al-Jarrah et al., 2015). Thus, there is an increasing demand for high-performance data inference tools that are both computationally cheap and not overly restrictive. Another main issue of several ML methods, such as deep learning, is the lack of inherent understanding of the rules underlying the learning during the data inference process (Rudin, 2019; Papernot et al., 2017). This is an obstacle for controlled and human-understandable improvement of the emerging methods (Deng et al., 2020). ML has strong links to information theory (such as the Shannon entropy, (log-) likelihood, and information content), which borrows many concepts of theoretical physics, particularly thermodynamics. The main goal of computing tools for data extraction is to (i) remove irrelevant data, usually associated with noise, and (ii) recognize patterns and behaviors in the underlying relevant data. These two processes can be carried out in a multitude of ways with tools that diverge, as follows, on their way to analyze and interpret the data. Concerning the first process, there are denoising tools (Buades et al., 2005; Shao et al., 2013; Goyal et al., 2020). For the second process, typically one assumes a model for the data such that one can infer latent behaviors. Some models are very powerful and broadly used such as Gaussian mixture models (GMMs) or hidden Markovian models (HMMs). However, imposing a model implies assumptions on the data and constrains information extraction. The most frequent assumptions are the identical and independent distribution (i.i.d.), as well as, Gaussianity of the data. Recently, several physics-inspired models have been proposed in order to take into account assumptions that best fit the observed dynamical systems (Williams et al., 2015; Runge et al., 2015; Ye et al., 2015; Vesselinov et al., 2019; Zenil et al., 2019; Rupe et al., 2019; Horenko et al., 2019). Furthermore, there has been a considerable effort in developing computationally scalable data-driven methods that allow overcoming bias imposed by the restrictive model assumptions of common tools (Gerber et al., 2020; Horenko, 2020). In particular, two novel physics-inspired methods—called latent entropy and latent dimension (Horenko et al., 2019)—not only combine both the denoising and pattern recognition step but also provide a computationally cheap measure for the data that allows for sharp recognition of patterns.

In this perspective, we discuss some state-of-the-art data-driven inference tools for analyzing latent effects in data and explain their applicability in natural science. In particular, in the Latent methods section, we will introduce (A) denoising tools, (B) model-based data analysis tools, such as GMMs, HMMs, and other physics-inspired models, and (C) two recently introduced, physics-motivated computationally cheap tools—the latent entropy and the latent dimension.

To exemplify the methods, in particular the latent entropy and latent dimension, in the Applications section, we first demonstrate them on a toy model clarifying the role of the latent entropy as a measure for stochasticity of the underlying dynamic process and the role of the latent dimension as a measure for the system's memory. Then, we apply them to (A) the data-driven visualization of the glymphatic system of a mouse brain from the in vivo brain microscopy data, revealing latent bulk capillaries; (B) an analysis of magneto-optical Kerr effect (MOKE) data from magnetization experiments, revealing a hidden gradient across the sample not visible to common measures; and (C) the analysis of two noisy astrophysical videos revealing features of the M57 (Ring Nebula) in Lira and the M31 star cluster in Hercules.

### Latent methods

Experimental measurements are typically performed by probing a sample via a perturbation and observing its response, which e.g., in microscopy amounts to shining light onto the sample and measuring how the sample reflects the light. Therefore, the quantity of interest can often only be captured indirectly through, for example, changes in the incident ray, photon counts, or event rates. The challenge is then to extract the desired information from the data acquired, which are typically affected by other physical processes as well as by noise. There is a major effort in finding the most appropriate methods to access and extract these latent—or hidden—phenomena of the observed system. The latent methods of modern information theory typically examine the statistical properties of the data (Bayes, 1763; Shannon, 1948; Tanner, 2012; Enβlin et al., 2009), inspired by the concepts like entropy—originally coming from statistical physics. In this perspective, we focus on the problem of spatial pattern recognition in video data.

### Denoising tools

Denoising tools remove unwanted identifiable noise thereby, allowing for a clearer comprehension of the relevant data (Buades et al., 2005; Shao et al., 2013; Goyal et al., 2020). The main challenge is to eliminate noise without compromising the relevant data. The methods vary greatly from local and non-local filters based on the correlation between pixels (Wiener, 1950; Brailean et al., 1995; Stockman and Shapiro, 2001; Buades et al., 2005; Shao et al., 2008) to mapping the data to other domains where patterns can be recognized (Dabov et al., 2007; Mairal et al., 2009; Luisier et al., 2010). To identify the noise, denoising tools typically require a model for the noise. With this purpose, they assume, for example, an oscillatory behavior or Gaussianity of the noise (Ernst, 1966; Wink and Roerdink, 2004; Luisier et al., 2011; Zhang et al., 2017). Some of the recent approaches attempt to learn a model for the noise based on ML tools (Hinton, 2006). In general, once the features of the data are classified within the model for the noise, the filter removes information that is considered unwanted, such as features with high frequency or that have low probability.

Denoising has become such a common instrument that it has been integrated in most mathematical software. In the section concerning the data analysis of the Glymphatic system of a mouse brain we have, for example, we use the denoising autoencoder of MATLAB (Zhang et al., 2017; Berghout et al., 2020, Berghout, 2021). In particular, this modified denoise autoencoder (Bengio and LeCun, 2007) is based on a supervised ML algorithm which models noise based on a training set. A filter is then used to remove the features from the data that correspond to the modeled noise.

Even though denoising tools can enhance the visualization of some data features by removing irrelevant information, they are unable to identify latent behaviors in the data.

### Model-based data analysis tools

Several model-based data analysis tools have been inspired by information theory and physics principles in order to extract relevant features from the data. Ideally, latent methods not only detect patterns in data, i.e., accurately group together pixels in video data with similar properties, but also are cost effective in terms of computational power. Below we present a few examples.

#### Gaussian mixture models

GMMs have been successfully implemented in a vast body of research fields, including economics (Barndorff-Nielsen and Shephard, 2001; Phillips and Loretan, 1991), genetics (François and Durand, 2010; Loh et al., 2015; Turelli and Barton, 1994), psychology (Bauer, 2007; Hipp and Bauer, 2006; Rachuri et al., 2010), and speech recognition (Torres-Carrasquillo et al., 2002; Reynolds, 1995). They assume that the underlying effects represented by the data are generated from Gaussian distributions characterized by a certain parameter set (Pedregosa et al., 2011; Melnykov and Maitra, 2010). The mean, covariance, and prior probability associated with each Gaussian distribution manifest the presence of latent variables. These parameters can be calculated by different variants of the expectation maximization algorithm (Frühwirth-Schnatter, 2006; Greggio et al., 2012; Pinto and Engel, 2015) which fits the mixture of Gaussian models that best identifies clusters in the analyzed data.

An important characteristic of GMMs is the invariance under reordering or permutation of the data sequence. The key assumptions of GMMs are the independency and identical distribution of the random variables (i.i.d. assumption), as well as homogeneity of the input data. The first two assumptions allow to fit properties of the data by a mix of Gaussian distributions, while the third assumption allows for fitting the data of all the frames with the same number of Gaussian distributions. These properties allow data to be analyzed for each frame independently and to be fitted by a mix of Gaussian distributions. GMMs are thus useful to track objects even when they are absent or occluded in some frames (McKenna et al., 1998; KaewTraKulPong & Bowden, 2002; Stauffer and Grimson, 1999; Santosh et al., 2013).

Below in the data analysis of images of the Glymphatic system of a mouse brain, we show how the GMM entropy allows us to recognize surface lymph capillaries from microscopy data of a mouse brain.

#### Hidden Markov models

HMMs take into account the time sequence of the frames to extract latent—“hidden”—information. They have been successfully applied to economics (Hassan and Nath, 2005; Dias et al., 2015), genetics (Collier et al., 2000; Stanke and Waack, 2003; Xi et al., 2010; Narasimhan et al., 2016), cancer diagnosis (Manogaran et al., 2018), speech recognition (Schuller et al., 2003), and weather research (Bellone et al., 2000). Their restrictive assumption is that the system is assumed to be a time-homogeneous Markov model such that each data frame depends solely on the previous via a stochastic process. More specifically, HMMs assume that the sequence of observables is obtained from a Markov process involving hidden states. By calculating the transition probability between the hidden states and the state observation likelihood from the hidden states, one obtains a model that is capable to predict outputs from given observables (Jurafsky and Martin, 2009). Physical systems, however, often present a more complex dynamics that cannot be captured by the time-homogeneous Markov model assumption.

#### Physics-inspired models

Physical principles can be leveraged to extract relevant information from data. By incorporating expected physical behaviors as basic assumptions into developing ML tools, it is possible to obtain predictive models for certain physical systems (Williams et al., 2015; Runge et al., 2015; Ye et al., 2015; Vesselinov et al., 2019; Zenil et al., 2019; Rupe et al., 2019). These models analyze the time evolution of the data features to obtain powerful predictive models that take into account subtle hidden behaviors. So far, however, most methods remain as conceptual contributions and algorithmic frameworks (Williams et al., 2015; Vesselinov et al., 2019; Zenil et al., 2019; Rupe et al., 2019). Implementations have been limited to certain physical systems, such as weather science (Runge et al., 2015; Ye et al., 2015; Rupe et al., 2019).

#### Scalable probabilistic approximation

A recent proposal toward a realistic implementation without suffering from overly restrictive assumptions from the models is the scalable probabilistic approximation (SPA) algorithms for complex systems (Gerber et al., 2020; Horenko, 2020). SPA algorithms are able to jointly solve clustering, feature selection, and Bayesian model inference problems for data analysis—providing discrete regular clustering approximations that are optimal for Bayesian prediction and classification, with a linear scaling of computational cost and with a parallel communication cost proven to be independent of data size (Gerber et al., 2020; Horenko, 2020). The mathematical properties of the obtained optimal SPA solutions (like regularity) have been demonstrated. The suboptimality of the clustering discretizations obtained with common methods like HMMs when compared to SPA, both in terms of quality and cost, has been proven mathematically. An important element to successfully implement a model is the ability to classify the data features according to a quantitative parameter, i.e., a measure. A measure can be used to cluster the data as well as to obtain insights into the observed system. For this reason, in the following, we focus on two recently introduced measures.

### Data measures: latent entropy and latent dimension

The latent entropy and latent dimensions are physics-inspired ML tools for reliable pattern recognition in video data (Horenko et al., 2019). Their algorithms incorporate thermodynamical principles to encode latent properties of the underlying system into two quantitative measures. They have been successfully implemented to analyze several systems, with different levels of complexity and spatiotemporal scales. It has been demonstrated that these tools detect even subtle patterns in data, such as revealing differences of up to 1% in material parameter change in a two-dimensional Ising model even far above the critical temperature (Horenko et al., 2019). They remain accurate also for large noise to signal ratios and are computationally cheap.

**Figure 1:**
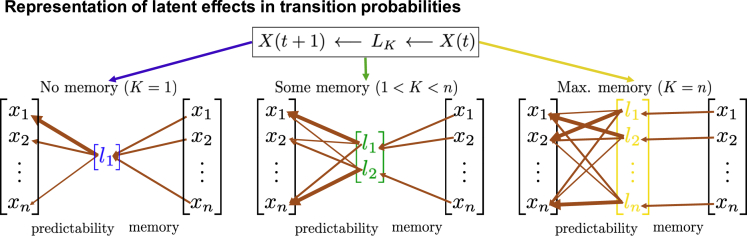
A representation of the meaning of the latent tools The transition to *X*(*t*+1) from *X*(*t*) may depend on hidden variables *L*_*K*_ with latent dimension *K*. The dependence between *X*(*t*+1) and *X*(*t*), i.e., memory of the system, is related to the dimension *K*. For example, if *K* = 1, the initial state *X*(*t*) is irrelevant and the system has no memory. The distribution of probabilities $P\left[X\left(t+1\right)=x_{j}|L_{K}=l_{k}\right]$, represented by the thickness of the arrows, contains information about the predictability of the system.

The two latent tools operate beyond the sometimes restrictive i.i.d. and Gaussianity assumptions, while taking into account the temporal ordering of the data to infer possible imprints of latent dynamic processes. Unlike GMMs which cluster data points by identifying common properties within each frame, the latent entropy and latent dimension take into account the dynamical evolution between frames to identify patterns in the data. The latter accesses more directly dynamical patterns such as density flows (see section on the Glymphatic system of a mouse brain) and change in the underlying physical parameters (see section on temperature gradient in magnetization experiment). The temporal ordering can be examined in terms of memory and predictability, i.e., the correlation between an initial and final state and the tendency of a system to remain in a set of states given the initial conditions, see Figure 1. The system's memory is characterized by the latent dimension while the latent entropy encodes the system's predictability. The sharp detection of latent processes for each pixel then allows for an accurate identification of patterns in the video data when considering the full spatial image.

We consider that each pixel at a time instance *t* can be described by *X*(*t*), an *n*-dimensional Euclidean vector. The components of *X*(*t*) are given by the probabilities $P\left[X\left(t\right)=x_{j}\right]$ of the pixel to assume a value *x*_*j*_ among *n* categories {*x*_1_,*x*_2_,⋯*x*_*n*_}. These *n* categories correspond to the discretized color scale of the pixel value. The time evolution of each pixel in time is, thus, given as a categorical sequence *X* = {*X*(*t* = 1),*X*(*t* = 2), …,*X*(*t* = *N*)}. The probability for *X* to attain a certain category at a certain time is given by the “exact law of the total probability” (Gardiner, 2004):

(Equation 1) $$P\left[X\left(t+1\right)=x_{j}\right]=\overset{n}{\underset{i=1}{\sum }}{\left({\text{Λ}}_{K}\right)}_{ji}P\left[X\left(t\right)=x_{i}\right],$$

where ${\left({\text{Λ}}_{K}\right)}_{ji}$ is the transition matrix describing the transition from the initial pixel state to its consecutive one through a potential latent process *L*_*K*_

(Equation 2) $${\left({\text{Λ}}_{K}\right)}_{ji}\equiv \overset{K}{\underset{k=1}{\sum }}P\left[X\left(t+1\right)=x_{j}\left|L_{K}=l_{k}\right]P\left[L_{K}=l_{k}\right|X\left(t\right)=x_{i}\right].$$

Here, $P\left[Z=z_{i}|W=w_{j}\right]$ denotes the conditional probability of *Z* assuming *z*_*i*_ while *W* is assuming *w*_*j*_. The latent process *L*_*K*_(*t*) takes values from *K* latent categories *l* = {*l*_1_,*l*_2_, …,*l*_*K*_} where *K* can assume any value between (1, …,*n*) (Hofmann, 1999, 2001), see Figure 1. In the following, we will use the shorthand notation ${\left(\lambda_{K}\right)}_{jk}=P\left[X\left(t+1\right)=x_{j}|L_{K}=l_{k}\right]$ and ${\left(\gamma_{K}\right)}_{ki}=P\left[L_{K}=l_{k}|X\left(t\right)=x_{i}\right]$ such that ${\text{Λ}}_{K}=\lambda_{K}\gamma_{K}$. Given this setting, the algorithm to compute the latent entropy and latent dimension as described in the study by (Horenko et al., 2019) is then given as follows (The MATLAB code is available on https://www.dropbox.com/s/w3few6elo9soegz/MATLAB_Code.zip?dl=0).

• Step 1: Compute the transition matrices ${\text{Λ}}_{K}$ based on the direct Bayesian model reduction (Hofmann, 1999, 2001; Ding et al., 2006; Gerber & Horenko, 2015, 2017; Gerber et al., 2018), as well as the quantities $S_{K}=-\frac{1}{N}\text{log}{\text{Λ}}_{K}$ for every *K* going from 1 to *n* (To be precise, $S_{K}=-\sum_{i=1}^{n}\sum_{j=1}^{n}C_{ij}\text{log}\left[{\left(\lambda_{K}\gamma_{K}\right)}_{ij}\right]$, where $C_{ij}=\frac{1}{N}\sum_{t=1}^{N}\chi \left(Y\left(t\right)=y_{i}\right)\chi \left(X\left(t\right)=x_{j}\right)$ with χ being an indicator function, is the average contingency table of the data *X* and *Y*.).
• Step 2: Determine the posterior probabilities *p*_*K*_ for the different latent dimensions *K* = 1, …,*n* by means of the Akaike information criterion (Hurvich and Tsai, 1989) as follows:

(Equation 3) $$p_{K}=\frac{\text{exp}\left(-\left(AICc_{K}-{\text{min}}_{K}AICc_{K}\right)\right)}{\sum_{K=1}^{n}\text{exp}\left(-\left(AICc_{K}-{\text{min}}_{K}AICc_{K}\right)\right)},$$

where $AICc_{K}=NS_{K}+V_{K}+\frac{V_{K}\left(V_{K}+1\right)}{N-V_{K}-1}$ and $V_{K}=\text{dim}\left(\lambda_{K}\right)-K+\text{dim}\left(\gamma_{K}\right)-n=\left(n-1\right)K+n\left(K-1\right)$.

• Step 3: Compute the average latent entropy and the average latent dimension as the following expectation values:

(Equation 4) $$\bar{S}=\overset{n}{\underset{K=1}{\sum }}p_{K}S_{K},\;\text{and}\;\bar{K}=\overset{n}{\underset{K=1}{\sum }}p_{K}K.$$

The latent dimension and latent entropy are, thus, calculated by assuming that the transition *X*(*t*)→*X*(*t*+1) may happen through any number of latent dimensions, *K* = 1,⋯*n*, and then weighting them with their posterior probability to occur. This can be seen in analogy to the concept of path integrals where the transition amplitude of a particle from an initial to a final state is computed as a weighted sum of all possible trajectories (Feynman, 1948; Feynman et al., 2010; Kleinert, 2009).

The average latent dimension K¯ quantifies how many latent states best describe each pixel's underlying dynamics. The smallest number of latent variables is 1, which corresponds to no memory at all, i.e., it does not matter which is the initial state since they are all taken to a single latent intermediate state. A higher K¯ indicates a higher dependence on the initial states. The average latent entropy S¯ quantifies the stochasticity of each pixel's underlying dynamics. A low S¯ means that the system is very predictable. A higher S¯ is associated to the increase in the randomness of the underlying dynamics.

The latent measures are calculated for each pixel individually; therefore, the iteration step in the calculation depends neither on the pixel number nor on the statistical size *N* as long as *N* > *n*^2^. The computation and memory cost depend only on the maximal number of latent dimensions *n* and scale as $O\left(n^{4}\right)$ and $O\left(n^{2}\right)$, respectively. For comparison, the computational costs of the GMM methods mentioned in the Gaussian mixture model subsection, for a given observational data of dimension *D* with *N* time frames and *n* possible Gaussian distributions (i.e. latent states), are to the leading order as $O\left(n^{2}ND\right)$ and require O(n(N+D)) of memory (Horenko et al., 2019).

We would like to note that this method works not only for considering each pixel individually but also when joining pixels into pixel patches. While bigger patches allow a deeper analysis of latent processes and long-range interactions, they cause loss of accuracy for pattern recognition. For example, joining all pixels to one block would remove the possibility to recognize any patterns. For each case, considering the data acquisition's sensitivity and resolution, there is an optimal pixel patch size. Nevertheless, analyzing the pixels independently is accurate for strong local interactions, particularly when each pixel can assume various values.

In the following, we will present a minimal model that explains the physical ideas of the latent entropy and latent memory.

#### Minimal model—a linear Markovian falling toast model

As a toy example, let us consider the study of “Murphy's law”, for which Robert Matthews obtained the IG Nobel Prize in 1996 (Matthews, 1995). In particular, Matthews demonstrated that it is not by implementation of the mysterious Murphy's law that a toast falls mostly on the buttered side, but it is instead a consequence of the latent effect of the standard table height from which the toast is falling. This is reflected in what we refer in this article as the average latent dimension K¯ (Equation 4). It encodes information about the degree to which the outcome depends on the initial configuration. As the typical table height influences the outcome, this means that the latent dimension is larger than one, i.e., the final state has a strong memory of the initial state. Generally, however, a fully symmetric toast falling from a sufficiently high table has on average the same chance of falling on either of the sides when sampling various starting configurations. This represents a maximal latent entropy S¯ (Equation 4) configuration. The latter can be reduced by favoring one of the sides, e.g., by spreading butter on one side. To make the notation used in the previous section even more explicit, we will describe the falling toast experiment in terms of its corresponding linear Markovian model.

**Figure 2:**
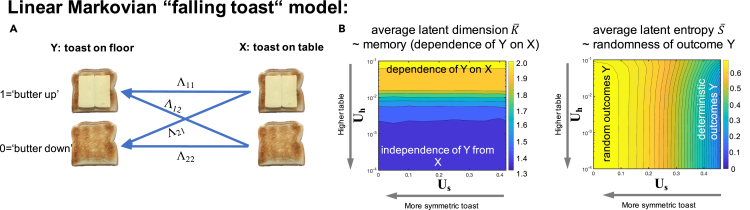
An explanatory toy example of the falling toast experiment (A) Visualization of transition probabilities between initial and final state; (B) Results for the latent dimension and latent entropy for different values of the two parameters of the model corresponding to the toast's asymmetry *U*_*s*_ and table heigth *U*_*h*_.

In this experiment, the initial and final states, *X*(*t* = 0)≡*X* and *X*(*t* = 1)≡*Y*, respectively, correspond to the orientation of the toast before and after falling from the table. The two possible configurations for *X* and *Y* are “butter up” which we will label by 1 and “butter down” which we will label by 0, see Figure 2A. The transition probability is given as follows:

(Equation 5) $$\left[\begin{array}{l} P[Y=1]\\ P[Y=0] \end{array}\right]\equiv \text{Λ}\left[\begin{array}{l} P[X=1]\\ P[X=0] \end{array}\right],$$

where Λ is the transition matrix given as follows:

(Equation 6) $$\text{Λ}=\left[\begin{array}{ll} P[Y=1|X=1] & P[Y=1|X=0]\\ P[Y=0|X=1] & P[Y=0|X=0] \end{array}\right].$$

According to the law of total probability, the columns of the transition matrix sum to 1. This implies that there are only 2° of freedom for this 2 × 2 matrix. We assign to these degrees of freedom the quantities *U*_*s*_ and *U*_*h*_ as deviations from the transition matrix corresponding to the Bernoulli model, where all probabilities are equal to 0.5, i.e.,

(Equation 7) $$\text{Λ}=\left[\begin{array}{ll} 0.5-U_{s}+U_{h} & 0.5-U_{s}-U_{h}\\ 0.5+U_{s}-U_{h} & 0.5+U_{s}+U_{h} \end{array}\right].$$

The parameter *U*_*h*_ (*h* for height) controls the randomizing effect due to the height of the table—as well as the amount of memory of the variable *Y* (toast on the floor) and its dependence on the variable *X* (toast on the table). The parameter *U*_*s*_ (*s* for symmetry) models a change of the asymmetry of the toast. Note that only combinations of parameters *U*_*h*_ and *U*_*s*_ are allowed such that all matrix entries are between zero and 1.

We consider three limiting cases to better understand the roles of *U*_*h*_ and *U*_*s*_.

1. For a symmetric toast falling from a high table, we have that Uh = Us = 0; therefore, the following equation is obtained:

(Equation 8) $$\text{Λ}=\left[\begin{array}{ll} 0.5 & 0.5\\ 0.5 & 0.5 \end{array}\right].$$

In this case, it is impossible to predict which face of the toast faces up when at the floor, i.e., it is independent of the initial state and both outcomes are equally probable. Thus, the system has no memory (K¯ = 1) and no predictability (S¯ is maximum). This corresponds to the Bernoulli experiment.

2. For a symmetric toast falling from a very low table (such that the toast has no chance to flip), we have that *U*_*s*_ = 0 and *U*_*h*_ = 0.5; therefore, the following equation is obtained:

(Equation 9) $$\text{Λ}=\left[\begin{array}{ll} 1.0 & 0.0\\ 0.0 & 1.0 \end{array}\right].$$

In this system, given an initial state, we know for sure the final state, i.e., the memory is maximal (K¯ is maximum, here K¯ = 2) and low predictability (S¯ is maximum).

3. For a very asymmetric toast falling from a high table, we have that *U*_*s*_ = 0.5 and *U*_*h*_ = 0; therefore, the following equation is obtained:

(Equation 10) $$\text{Λ}=\left[\begin{array}{ll} 0.0 & 0.0\\ 1.0 & 1.0 \end{array}\right].$$

Asymmetric toast means that the center of mass is shifted in such a way that the toast tends to have the buttered face down while the high table means that it has time to flip, if necessary. The final state is, therefore, independent of the initial state. This means that the system has no memory (K¯ = 1), but high predictability (S¯ = 0), as the outcome is clear.

After 100 random experiments according to the model in Equation 7, for every combination of the parameters *U*_*s*_ and *U*_*h*_, we calculated the expected values of the average latent entropy and average latent dimension, see Figure 2B. We find that the average latent dimension depends only on the parameter *U*_*h*_ while it is independent of *U*_*s*_, whereas for the latent entropy, it is the other way around. This can be understood in the following way: the parameter *U*_*h*_ determines the correlation between an initial and a final state, i.e., the memory of the system. The smaller *U*_*h*_ the more deterministic is the system's behavior, meaning the fewer latent processes appear and thus the smaller the latent dimension. Increasing *U*_*s*_, on the contrary, increases the predictability of the outcome and therefore leads to a smaller latent entropy, while not influencing the system's memory. Figure 2B indicates that the latent dimension and latent entropy are two rather orthogonal measures revealing very different types of information content.

## Applications

In this section, we will exemplify the strength of latent measures, focusing on the latent entropy, by presenting the analysis of data from three different fields in natural science belonging to different space and time scales. First, we apply the methodology to biological systems, specifically the microscopy video of a lymph flow in a mouse brain. We demonstrate that the latent entropy measure reveals previously poorly observed parts of the glymphatic network system (Iliff et al., 2012; Begley, 2012). We will then present an application to analysis of video data from micromagnetic experiments, revealing the non-trivial latent features in the underlying sample. We show that the high accuracy in examining the underlying dynamics allows for detecting latent entropy gradients in the magnetic samples. In the analyzed data, we associate the detected gradient patterns to a variation of temperature along the sample. As a third example, we apply the latent tools to amateur astronomical observations to demonstrate that even with low sensitivity of the particular astronomic instruments and significant atmosphere-induced noise, it is possible to detect subtle features that are otherwise only directly accessible to much more powerful instruments.

### Glymphatic system of a mouse brain

**Figure 3:**
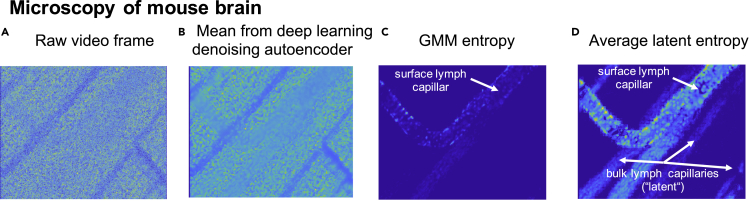
Analysis of the microscopy video of lymph flow in a mouse brain (A–D) (A) Raw video frame, (B) results obtained by calculating the mean from the deep learning denoising autoencoder, (C) analysis by means of the GMM entropy clearly revealing surface lymph capillaries, (D) analysis by means of the latent entropy revealing even bulk lymph capillaries.

In this first example, we show the power of the latent measure to observe the glymphatic system of a mouse brain. The glymphatic system in the brain was unobserved for a long time because the fluid in the glymphatic network is transparent. Until 2012, there was no solid evidence for its existence (Iliff et al., 2012). With the use of advanced latent measures, however, one can visually observe the flux corresponding to the glymphatic network from microscopic video of a mouse brain, see Figure 3.

The glymphatic system constitutes an autonomous lymph transport network in the brain, responsible for the disposal of waste products during the sleeping phase (Iliff et al., 2012; Begley, 2012; Nedergaard, 2013; Benveniste et al., 2019). Deeply understanding its functionality is particularly important in the study of neurodegenerative diseases, which are associated with an atypical accumulation of cellular waste products (Benveniste et al., 2019).

Figure 3 summarizes the analysis results for the light microscopy video of the living mouse brain tissue with a flow of the transparent lymph fluid through the capillaries of the glymphatic system. The capillaries of the glymphatic system are not directly visible either in the raw video data, see Figure 3A, or in the mean obtained by using a commercial deep learning denoising autoencoder from the “Deep Learning Toolbox” of MATLAB (Zhang et al., 2017; Berghout et al., 2020, Berghout, 2021), see Figure 3B. Application of the common GMM entropy (Zoran and Weiss, 2011; Bouman et al., 2018; The Event Horizon Telescope Collaboration, 2019a; Greggio et al., 2012) does allow to visualize only surface capillaries, see Figure 3C. The deeper lying bulk capillaries are not directly visible and can only be revealed through latent effects. As can be seen from the Figure 3D, applying the latent entropy measure one can extract and visualize the capillary pattern, including the bulk ones.

### Temperature gradient in magnetization experiment

**Figure 4:**
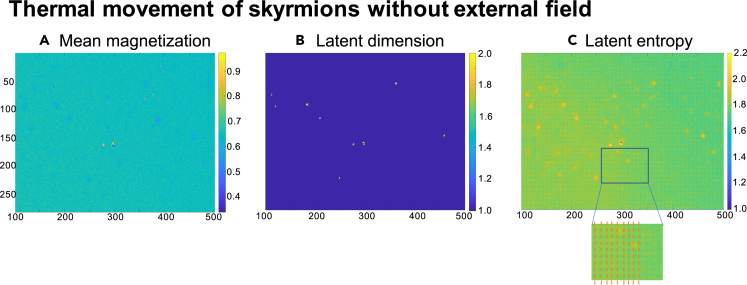
Data analysis of a MOKE experiment The axes denote the number of pixels and the color code represents (A) the mean value of the out-of-plane magnetization, (B) the latent dimension, and (C) the latent entropy. The inset elucidates the superstructure grid pattern imposed by the video compression.

In this second example, we show that the high sensitivity of the latent measures allows detecting subtle material inhomogeneities by analyzing the video data from micromagnetic measurements. We apply the latent tools to a video of the magnetization configuration in specially tailored low-pinning, multilayer material Ta(5nm)/Co_20_ Fe_20_B_20_(1nm)/Ta(0.08nm)/MgO(2nm)/Ta(5nm) stacks (Zázvorka et al., 2019) obtained by MOKE microscopy (Hubert & Schäfer, 2009; Huang et al., 1994). In this experiment, for each resolution pixel, only the out-of-plane component of the magnetization is detected at about room temperature (Note that 305 pixels correspond to 50 *μm*.). The time step between two measurements, i.e., two video frames, is 62.5 ms. The experiment in Figure 4 aimed at studying rather homogeneous materials, striving for a free motion of magnetic skyrmions and avoiding impurities where magnetic textures get pinned. The time record of the skyrmions' positions, however, revealed that there are preferred positions where the skyrmions tend to stay longer and which can indirectly be associated to the existence of inhomogeneities (Zázvorka et al., 2019). These inhomogeneities strongly influence, for example, the temperature dependence of the skyrmion diffusion coefficient (Zázvorka et al., 2019), and thus, it is important to detect even weak or small material defects. Strong material inhomogeneities can be resolved by simple means such as the mean value of the magnetization, as shown in Figure 4A. The average latent dimension (see Figure 4B) sharply identifies impurities where skyrmions are more likely to be pinned, e.g., where the skyrmions have a longer memory of their previous state. The latent entropy (see Figure 4C) not only reveals material inhomogeneities as spots of higher entropy but also allows us to discern two other significant features: an entropy gradient across the sample and a subtle grid pattern superstructure, as shown in the inset. The latter is induced by the video compression applied to the experimental data, as discussed in the study by (Horenko et al., 2019).

The gradient in the latent entropy is associated with a field gradient in the underlying magnetization dynamics, and the experimental conditions are consistent with the interpretation that it corresponds to a temperature gradient in the sample. This opens up a path toward analyzing the temperature distribution across devices, which are typically very hard to detect by means of common magnetic imaging techniques.

### Astronomical observations

In this third example, we show how the latent tools can be used to extract latent features from noisy amateur astronomy videos. We apply the latent tools explained above to remove the noise induced by atmospheric fluctuations and to detect the underlying features by identifying the dynamical patterns in the video data.

**Figure 5:**
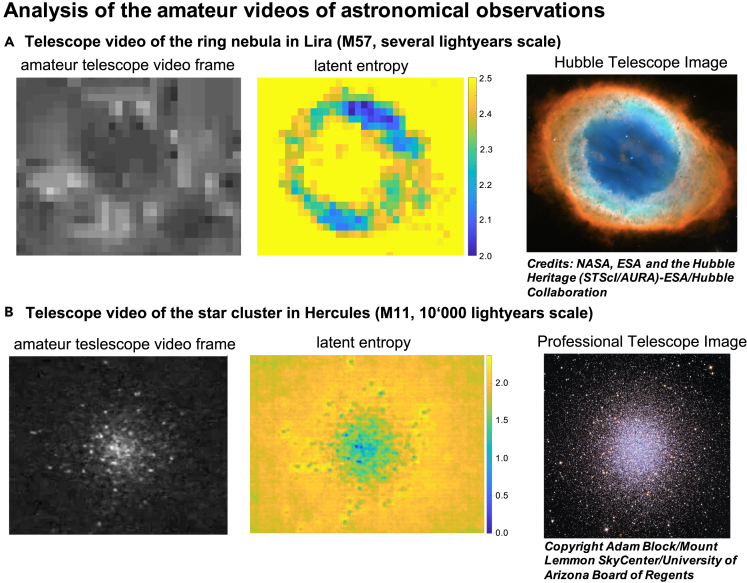
Analysis of the amateur videos of astronomical observations (A) for the M57 Ring Nebula in Lira and (B) for the M31 star cluster in Hercules.

Figure 5 shows results obtained for two amateur infrared videos: (a) Ring Nebula in Lira (M57) and (b) a star cluster in Hercules (M11), comparing the patterns obtained with the latent measures to the direct observations with the Hubble Space Telescope. We notice that despite of the apparent strong atmospheric fluctuations, these two videos can still be used to extract the underlying image patterns of a remarkable quality. Comparing the frames from the raw video data in the first column of Figure 5 with the latent entropy measure (middle column), one can see that the number of details, for instance, the star concentration, is significantly increased such that it becomes much closer to the features obtained with the Hubble telescope, as shown in the third column of Figure 5.

Computational and statistical methods to identify subtle features in the noisy video data are of large importance for astronomical observations and allowed for important discoveries in the past, such as the cosmic microwave background radiation, gravitational waves, existence and motion of exoplanets, and imaging of black holes (Zoran and Weiss, 2011; Greggio et al., 2012; Bouman et al., 2018; The Event Horizon Telescope Collaboration, 2019b). The high accuracy and the low computational cost of the latent measures may potentially help to enhance a detection of weak latent features in these application domains.

## Discussion and conclusion

We have shown the utility of advanced data analysis tools in application to problems of pattern extraction and denoising across a wide range of spatiotemporal scales, from nanoscales to astronomic length scales, discussing also potential consequences for disparate research fields ranging across physics and biology. Particular emphasis was placed on the recently introduced data analysis tools—on the latent entropy and the latent dimension—aiming at disentangling the latent effects induced by predictability and memory in the observed data dynamics. Operating beyond common assumptions like Gaussianity and homogeneity and taking into account the temporal ordering of the data, it was shown that these tools can reveal subtle latent features that are otherwise invisible to popular latent inference methods like GMMs and deep learning denoising autoencoders. In particular, these tools also avoid overfitting issues as they build an expectation over all possible non-parametric (i.e., without explicit parametric assumptions like Gaussianity) latent fits with latent dimension K spanning the whole range from *K* = 1 to *K*=*N*. The calculation of expectation values is based on the posterior Akaike weights—one of the popular tools of information theory that were designed to avoid overfitting.

A common caveat for all of the data-driven measures compared in this manuscript is that even though these methods can help to identify different patterns hidden in the data, they do not directly provide means to determine the precise sources of these different patterns. They do, however, reveal interesting and even subtle features and inhomogeneities that can then be further investigated.

To summarize, machine learning and data analysis tools will play an increasingly important role in various application areas—and exploiting advanced tools that aim at detecting latent dynamical patterns in time-resolved data promises to achieve much deeper insights into the nature of the underlying phenomena.
